# Supplementary material for: The Pharmacists of Physical Activity: Physiotherapists Empowering Older Adults’ Autonomy in the Self-Management of Aging with and Without Persistent Conditions
Source: Healthcare (Basel). 2025 Apr 6;13(7):834. doi: 10.3390/healthcare13070834 (PMC11988951; doi:10.3390/healthcare13070834)

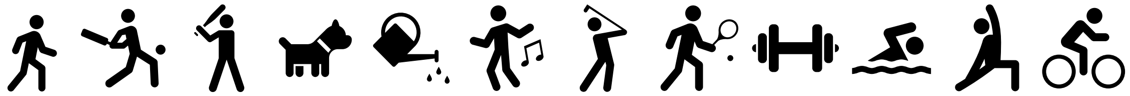

My Physical Activity Goal and Plan

Did you know that physical activity can help you do the things that are important to you and decrease your need for future physical therapy visits?

If you are interested in becoming more active, it can be helpful to figure out what your goals are, what type of activities you enjoy, and how you will do them. If you would find this helpful, please complete the below questions and bring it to your next physical therapy appointment. Try to be as specific as possible.

What are your goals?

(These can be related to physical therapy or physical activity.)

____

____

What type of physical activity would you like to do?

____

____

What is your Plan?

Where will you do it? ____________________________

When will you do it? ____________________________

How often will you do it? _________________________

How much will you do? __________________________

When will you start? ____________________________

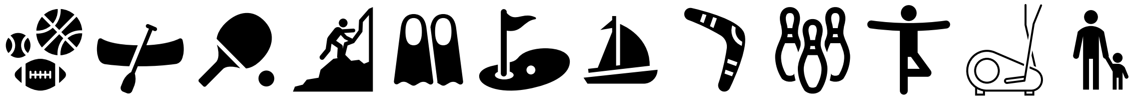

Supplement: Supplementary file 1 [file healthcare-13-00834-s001.zip › healthcare-3505269-supplementary.pdf]
